# Protective Mechanisms of Exosomes Derived From Human Umbilical Cord Mesenchymal Stem Cells in Bronchopulmonary Dysplasia

**DOI:** 10.33549/physiolres.935542

**Published:** 2025-06-01

**Authors:** Shu-Hang CAI, Li YANG, Xiao-Jie HE, Qiu-Yue ZHANG

**Affiliations:** 1 Department of Pediatrics, The First Affiliated Hospital of Hainan Medical University, Haikou, Hainan Province, China; 2 Department of Pediatrics, The Second Xiangya Hospital of Central South University, Changsha, Hunan Province, China

**Keywords:** Bronchopulmonary dysplasia, Exosomes, Human umbilical cord mesenchymal stem cells, Macrophages, NLRP3

## Abstract

Bronchopulmonary dysplasia (BPD) is characterized by reduced alveolar formation and disordered matrix remodeling. Currently, there are no effective therapeutic approaches for it. This study aims to explore the protective effect of exosomes derived from human umbilical cord mesenchymal stem cells on BPD by regulating the immune response and inflammatory pathways of macrophages. PKH26-labeled human umbilical cord mesenchymal stem cell line exosomes (hUCMSC-Exos) were co-cultured with RAW264.7 cells, which were assigned to the following groups: normoxia, normoxia + NLRP3 activator (Nigericin), normoxia + hUCMSC-Exos + Nigericin, hyperoxia, hyperoxia + hUCMSC-Exos, and hyperoxia + hUCMSC-Exos + Nigericin. Cell viability and cytokine expression in cell supernatant were measured for each group. PKH26 exosome staining confirmed successful uptake of hUCMSC-Exos by RAW264.7 cells. hUCMSC-Exos demonstrated protective effects against reductions in cell viability induced by both Nigericin and hyperoxia. Cells in the Hyperoxia group showed significantly increased expression of inflammatory cytokines IL-33, IL-6, IL-1β, TNF-α, and IL-18 compared to those in the Normoxia group, along with elevated mRNA and protein levels of NLRP3, ASC, caspase-1, IL-18, IL-1β, and ATF4. The Hyperoxia + hUCMSC-Exos group exhibited reduced expression of IL-33, IL-6, IL-1β, TNF-α, IL-18 and IL-33, IL-6, IL-1β, TNF-α, and IL-18 compared to the Hyperoxia group. In contrast, the Hyperoxia + hUCMSC-Exos + Nigericin group showed elevated levels of IL-33, IL-6, IL-1β, TNF-α, and IL-18, as well as increased expression of NLRP3, ASC, caspase-1, IL-18, IL-1β, and ATF4 compared to the Hyperoxia + hUCMSC-Exos group. hUCMSC-Exos mitigate hyperoxia-induced damage to lung macrophages by reducing endoplasmic reticulum stress, inhibiting NLRP3 inflammasome expression, and regulating inflammatory cytokine release, that may be potentially useful in BPD.

## Introduction

Bronchopulmonary dysplasia (BPD) is a prevalent pulmonary disorder in premature infants, characterized by impaired alveolarization and disruptions in matrix remodeling. These structural changes may persist beyond adolescence, significantly impacting the physical and mental health of pediatric patients [1]. Multiple factors contribute to BPD development, including genetics, oxygen toxicity, lung injury, infection, and malnutrition. Despite advancements in neonatal care over the past three decades, the incidence of BPD has remained relatively unchanged, with treatment outcomes still unsatisfactory [2,3]. This highlights the urgent need to understand its pathogenesis mechanism and develop new therapeutic approaches.

The immune system plays a pivotal role in the pathogenesis of BPD. Macrophages, as key components of the innate immune system, are present throughout the body’s tissues and participate extensively in biological processes, including immune responses, tissue repair, and maintaining homeostasis [4,5]. Dysfunction of macrophages contributes to the onset and progression of BPD. Studies have shown that both resident and non-resident macrophages respond to injury in inflamed lung environments by coordinating pulmonary inflammatory responses through the secretion of various cytokines, such as interleukin-6 (IL-6) [6]. Notably, the NLRP3 inflammasome acts as a pattern recognition receptor (consisting of NLRP3, the caspase recruitment domain, apoptosis-associated spot-like protein (ASC), and Caspase-1). Its abnormal activation has been shown to be closely associated with acute lung injury. When lung tissue is subjected to mechanical or metabolic damage, overactivation of the NLRP3 inflammasome can lead to a sustained inflammatory response [7–9]. The endoplasmic reticulum stress mechanism closely related to the NLRP3 pathway is also worthy of attention. Activating transcription factor 4 (ATF4) serves as the primary upstream regulator of cellular endoplasmic reticulum stress. By activating the ATF4-CHOP-GADD34/ERO-1α pathway, it induces oxidative stress and inflammation, which lead to the increased levels of inflammatory cytokines, such as IL-1, IL-4, and IL-8, thereby playing a crucial role in endoplasmic reticulum stress [10–12]. Studies have revealed that ATF4 expression is significantly upregulated following LPS-induced cellular endoplasmic reticulum stress and NLRP3 inflammasome activation. The endoplasmic reticulum stress inhibitor 4-PBA suppresses both endoplasmic reticulum stress and ATF4 expression while downregulating NLRP3 expression [13].

Based on the above mechanisms, researchers focused on human umbilical cord mesenchymal stem cell exosomes with immunomodulatory functions. Such extracellular vesicles have been proven to reshape the inflammatory microenvironment by regulating the phenotype of macrophages: In the pulmonary arterial hypertension model, hUCMSC-Exos can promote the polarization of M2-type macrophages and reduce the levels of IL-6, TNF-α and other pro-inflammatory factors [14–15]. Therefore, this study hopes to systematically explore the effects of hUCMSC-Exos on NLRP3 inflammasome activation and endoplasmic reticulum stress, as well as its mechanism in regulating macrophage function and inflammatory response by constructing an *in vitro* model of BPD (such as pulmonary macrophage model under high oxygen exposure). To verify that exosomes inhibit the expression of NLRP3, inhibit the release of inflammatory factors, and reduce the damage of macrophages in the case of hyperoxia, which may be one of the protective mechanisms against bronchopulmonary dysplasia.

## Materials and Methods

### Ethical approval

All applicable international, national, and/or institutional guidelines for the care and use of animals were followed.

### Materials

Mouse monocyte macrophage cell line RAW264.7 was obtained from Shanghai Donghuan Biological Technology Co., Ltd. The DMEM (Dulbecco’s Modified Eagle Medium) culture medium and 1 % double antibiotics were sourced from Suzhou Junxin Biotech Co., Ltd., while 10 % fetal bovine serum (FBS) was provided by Suzhou Shuangru Biotechnology Co., Ltd. Gene primers for NLRP3, ASC, caspase-1, IL-1β, IL-18, ATF4 were designed by Shanghai Donghuan Biological Technology Co., Ltd., and synthesized by Shanghai Generay Biotech Co., Ltd. Antibodies used included NLRP3 and rabbit secondary antibody (Abcam), ASC (ABclona), caspase-1 (Affinity), IL-1β (Proteintech), IL-18 (Immunoway), and Actin (ABways). Additional materials included the Cell Counting Kit-8, RNA extraction kit, reverse transcription kit, protein lysis buffer, ELISA detection kit, and ECL developer, all supplied by Shanghai Donghuan Biological Technology Co., Ltd.

### Cultivation and identification of hUCMSC-Exos

Human umbilical cord mesenchymal stem cells (Fuheng) were cultured and passed through. The exosomes in the cell supernatant were extracted according to the extractor kit. The cell culture supernatant was collected, centrifuged at 3000 RPM at 4 °C for 10 min, cell debris in the sample was removed, the cell culture medium was mixed with ECS reagent in a ratio of 4:1, left for 4 h, centrifuged at 10000 RPM at 4 °C for 60 min, and the supernatant was discarded. Subsequently, the obtained precipitate was re-suspended with 30 ml PBS solution and carefully mixed to ensure uniform dispersion of exosomes. This was followed by centrifugation at 6500 RPM for 70 min to further isolate and concentrate exosomes. After centrifugation, the obtained exosomes were finally suspended in 1 ml PBS solution, and the obtained exosome solution was stored in the refrigerator at −80 °C. The purified exosomes were identified by Western blotting analysis, NTA and TEM.

### Red fluorescent labeling of exosomes

Exosome protein concentration was determined using the bicinchoninic acid (BCA) method. A 100 μM dye working solution was prepared and protected from light. The dye solution was then added to the exosome sample at a ratio of 3:1 (exosomes to working solution). After thorough mixing and a 10-minute incubation, 10 ml of 1×PBS was added, and the mixture was further processed for exosome extraction. The final precipitate was resuspended in 200 μl 1×PBS resulting in red fluorescently labeled hUCMSC-Exos suitable for experimental use.

### Cell culture

RAW264.7 cells were cultured in DMEM culture medium supplemented with 10 % fetal bovine serum, maintained in a cell incubator at 37 °C with 5 % CO_2_. The culture medium was refreshed every 24 h. Once cell density reached 80 % confluence within the culture flask, cells were digested with trypsin and passaged.

### Cell grouping, plating, and treatment

The RAW264.7 cells were allocated into the following groups: Normoxia (21 % O_2_), Normoxia + Nigericin group (21 % O_2_, adding Nigericin), Normoxia + hUCMSC-Exos + Nigericin (21 % O_2_, adding hUCMSC-Exos, Nigericin), Hyperoxia (90 % O_2_), Hyperoxia + hUCMSC-Exos (90 % O_2_, adding hUCMSC-Exos), and Hyperoxia + hUCMSC-Exos + Nigericin (90 % O_2_, adding hUCMSC-Exos and Nigericin). Cell seeding details are provided in Table 1. (Note: All cells were cultured in a cell culture incubator at 37 °C and 5 % CO_2_. The cultivation time is 48 h).

### CCK-8 cell viability detection

Cells from each group were seeded into 96-well plates. A volume of 10 μl of cell counting kit-8 (CCK-8) solution was added to each well and incubated for 2 h. Absorbance at 450 nm was measured using a microplate reader to assess cell viability. Each group was tested in triplicate, and the mean absorbance values were calculated to determine cell proliferation activity at different time points.

### ELISA detection

Reagents, samples, and standards were prepared according to the manufacturer’s protocol. A volume of 50 μl of standards or samples was added to each well, immediately followed by 50 μl of Detection Reagent A. Plates were incubated at 37 °C for 1 h. Wells were washed three times with 200 μl wash solution, and plates were then dried. Subsequently, 100 μl of prepared Detection Reagent B was added to each well, and the plates were incubated at 37 °C for 30 min. After incubation, the wells were washed five times with 200 μl wash solution and dried again. A 90 μl volume of substrate solution was added, and the plates were incubated at 37 °C for 10–20 min, after which 50 μl of stop solution was added. The absorbance at 450 nm was measured immediately.

### Quantitative Real-Time PCR (qRT-PCR) analysis

Total RNA was extracted from each experimental group following the Trizol reagent kit protocol. RNA purity and concentration were measured using a UV spectrophotometer. Reverse transcription was performed based on the reverse transcription kit protocol, converting mRNA into complementary DNA (cDNA) using specific primer sequences, as detailed in Table 2. Furthermore, PCR amplification was performed using cDNA as a template to achieve amplification and analysis of target sequences.

### Western blot analysis

Proteins from each experimental group were separated by SDS-PAGE gel electrophoresis and transferred to membranes. After transfer, the membranes were blocked with 5 % skimmed milk powder for 2 h. Subsequently, membranes were washed three times in PBST solution, each wash lasting 10 min. Membranes were incubated overnight at 4 °C with specific primary antibodies. After incubation, the membranes were washed three times in PBST for 10 min each. The membranes were then incubated with a goat anti-rabbit secondary antibody for 2 h at room temperature. Following a final wash in PBST, protein signals were visualized and quantified using the electrochemiluminescence (ECL) technique. The gray values of target proteins were statistically analyzed. Details of the antibody dilution ratios are provided in Table 3.

### Statistical analysis

All the data collected in this study were analyzed using Prism 8.0 software. Normally distributed measurement data were expressed as mean ± standard deviation (SD), and the comparisons were examined by Student *t*-test. P<0.05 was considered statistically significant.

## Results

### Identification of hUCMSC-Exos

**Fig. 1:**
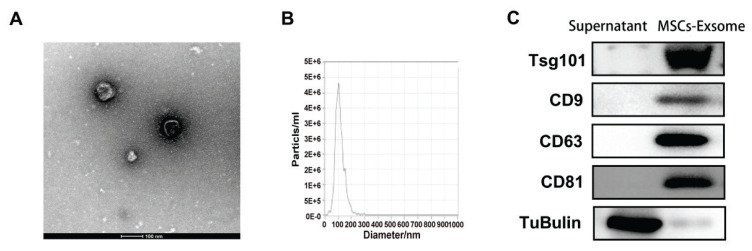
Identification of hUCMSC-Exos. (**A**) Transmission electron microscopy of exosomes (scale bar = 100 nm); (**B**) Detection of typical particle size of hUCMSC-Exos in submitted samples; (**C**) Immunoblotting detection of exosome markers.

HUCMSC-Exos were extracted from hUCMSC supernatant through ultracentrifugation. Transmission electron microscopy revealed that hUCMSC-Exos exhibited a cup-shaped morphology, a bilayer membrane, and a near-circular structure, as illustrated in Figure 1A. Nanoparticle tracking analysis measured particle sizes based on Brownian motion trajectories. Repeated measurements indicated that exosome particle size peaks were not completely identical. The diameter of hUCMSC-Exos samples for testing is mostly between 100 nm, as shown in Figure 1B. Surface markers of the hUCMSC-Exos, including CD9, CD63, CD81, and Tsg101, were identified through Western blot analysis, as depicted in Figure 1C. These findings validated that the extracted material consisted of exosomes derived from human umbilical cord mesenchymal stem cells.

### Labeling of hUCMSC-Exos

**Fig. 2:**
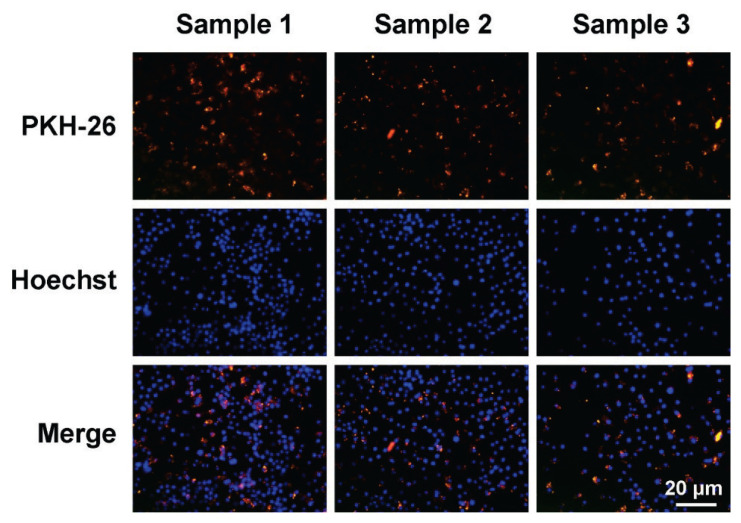
Exosomes uptake by RAW264.7 macrophages.

Exosomes were labeled with PKH26 red fluorescent dye and co-cultured with RAW264.7 macrophages. Results showed that macrophages successfully internalized exosomes across different samples, as illustrated in Figure 2.

### CCK-8 detection of cell viability in each group

**Fig. 3:**
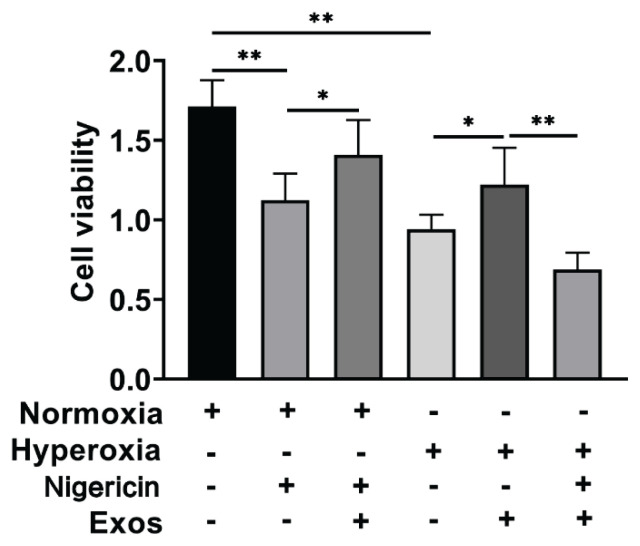
CCK-8 Detection of cell proliferation activity. Note: ** P<0.001, * P<0.05.

CCK-8 experiments demonstrated that hUCMSC-Exos mitigated the reduction in cell viability induced by Nigericin and hyperoxia conditions (P<0.05), as illustrated in Figure 3.

### qRT-PCR detection of NLRP3, ASC Caspase-1 and ATF4 expression across groups

**Fig. 4:**
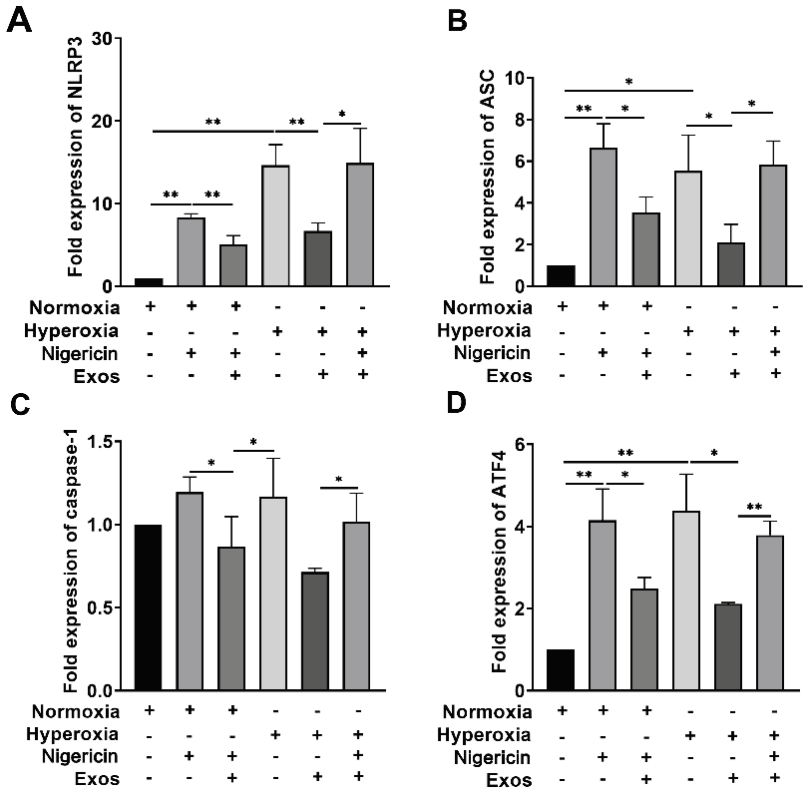
mRNA expression levels of NLRP3, ASC, Caspase-1 and ATF4. (**A, B, C** and **D**) mRNA expression levels of NLRP3, ASC, caspase-1 and ATF4 respectively, in monocyte macrophages. Note: ** P<0.001, * P<0.05.

Quantitative reverse transcription PCR (qRT-PCR) was conducted to assess the mRNA expression of components of the NLRP3, ASC, Caspase-1 and ATF4 across experimental groups. The results showed that compared with Normoxia group, mRNA expressions of NLRP3, ASC, caspase-1 and ATF4 in Hyperoxia group were increased. Compared with Hyperoxia group, mRNA expressions of NLRP3, ASC, Caspase-1 and ATF4 were increased. mRNA expressions of NLRP3, ASC, caspase-1 and ATF4 were decreased in Hyperoxia + hUCMSC-Exos group. Compared with Hyperoxia + hUCMSC-Exos + Nigericin group, mRNA expressions of NLRP3, ASC, caspase-1 and ATF4 were higher in Hyperoxia + hUCMSC-Exos + Nigericin group (Fig. 4).

### Western blot detection of NLRP3, ASC, Caspase-1 and ATF4 expression across groups

**Fig. 5:**
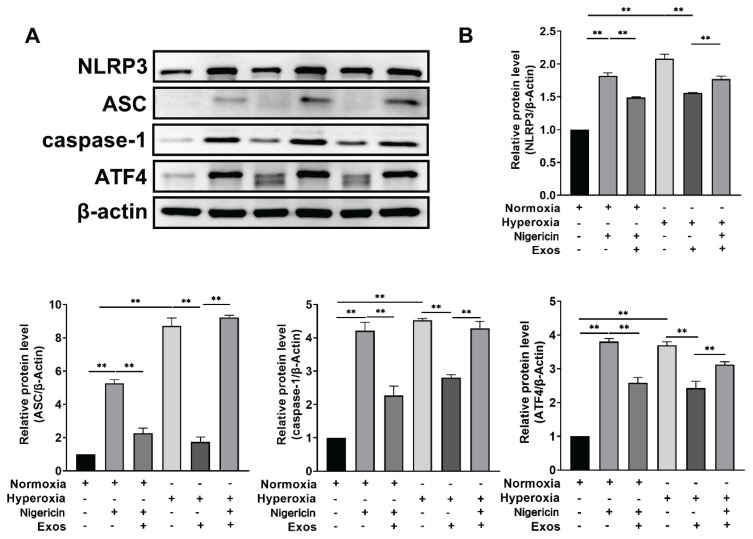
Expression of NLRP3, ASC, Caspase-1 and ATF4 in Monocyte Macrophages. Protein expression levels of NLRP3, caspase-1, ASC and ATF4 in monocyte macrophages, respectively. (**A**) Western blot images. (**B**) Quantitative bar graphs for each protein (NLRP3, ASC, Caspase-1, ATF4). Note: ** P<0.001, * P<0.05.

Western blot detection of NLRP3-related protein expression across the experimental groups demonstrated that, in comparison to the Normoxia group, the Hyperoxia group demonstrated significantly higher expression of NLRP3, ASC, caspase-1 and ATF4 (P<0.05). When compared to the Hyperoxia group, protein expression levels of NLRP3, ASC, caspase-1 and ATF4 were reduced in the Hyperoxia + hUCMSC-Exos group (P<0.05). Additionally, relative to the Hyperoxia + hUCMSC-Exos group, protein expression levels of inflammasome-related markers NLRP3, ASC, caspase-1 and ATF4 were increased in the Hyperoxia + hUCMSC-Exos + Nigericin group (P<0.05), as shown in Figure 5.

### ELISA detection of inflammatory factors in cell supernatant across groups

**Fig. 6:**
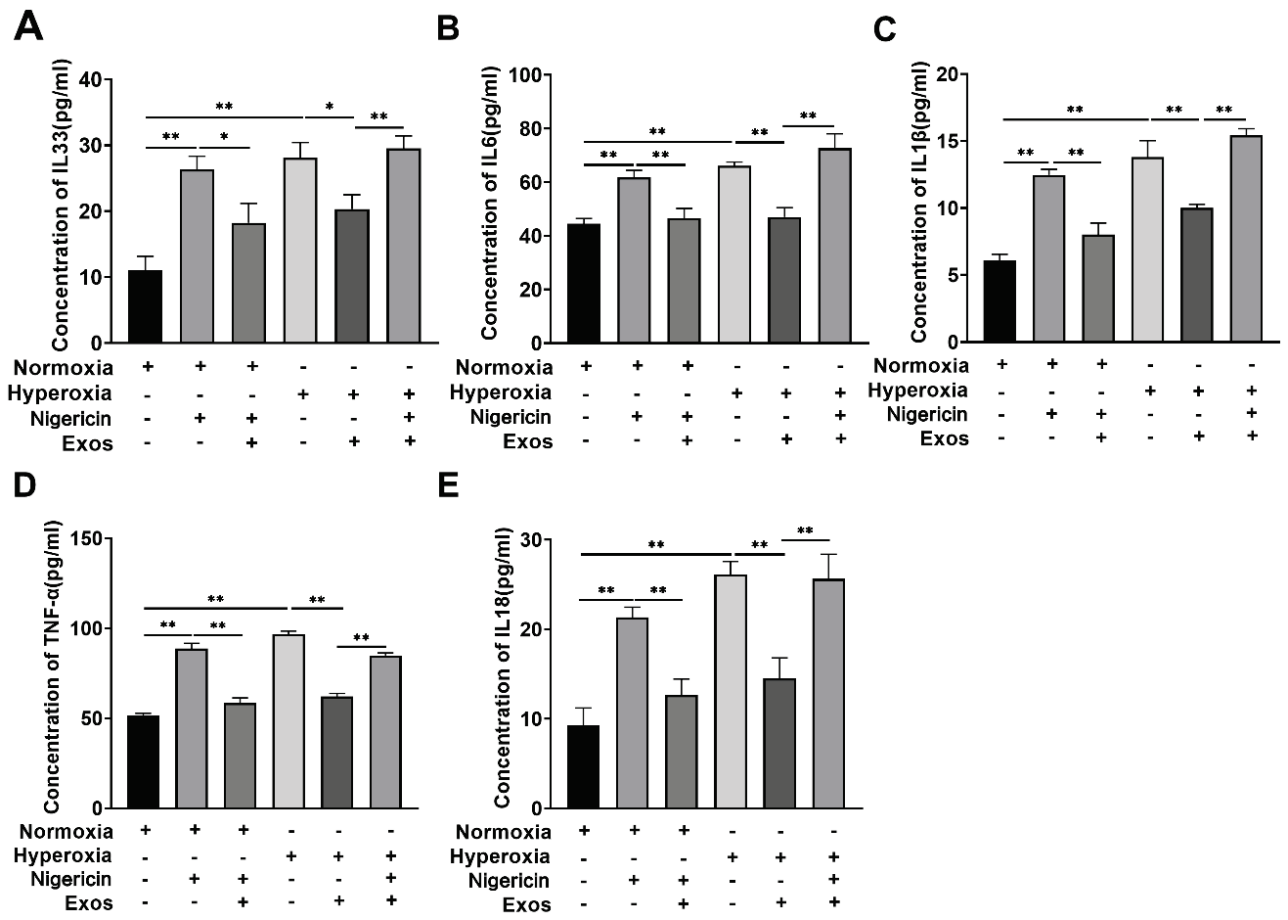
Levels of cell inflammatory factors. (**A, B, C, D**, and **E**) Levels of IL-33, IL-6, IL-1β, TNF-α, and IL-18 in monocytes macrophages, respectively. ** P<0.001, * P<0.05.

Enzyme-linked immunosorbent assay (ELISA) was performed to measure the levels of inflammatory cytokines in the cell supernatant across the experimental groups. Compared to the Normoxia group, the Hyperoxia group exhibited significantly elevated levels of IL-1β, IL-18, IL-6, IL-33, and TNF-α (P<0.05). The levels of these cytokines were significantly reduced in the Hyperoxia + hUCMSC-Exos group compared to the Hyperoxia group (P<0.05). However, when compared to the Hyperoxia + hUCMSC-Exos group, the Hyperoxia + hUCMSC-Exos + Nigericin group showed an increase in IL-1β, IL-18, IL-6, IL-33, and TNF-α levels (P<0.05), as illustrated in Figure 6.

## Discussion

Bronchopulmonary dysplasia (BPD) is a prevalent complication among preterm infants [16], affecting over 25 % of those with birth weight below 1500× g [17]. It is associated with prolonged hospitalization, increased mortality rates, and a higher likelihood of developing chronic pulmonary diseases. Infants with BPD experience elevated risks of chronic respiratory injury, cardiovascular impairment, and neurodevelopmental delays when compared to preterm infants without BPD. These long-term health challenges not only increase the medical burden but also adversely affect the quality of life and developmental outcomes for infants in clinical care [18].

However, the specific pathogenesis of BPD remains unclear, and effective treatments are still lacking. Alveolar macrophages play crucial roles in both lung tissue repair and remodeling, as well as in clearing pathogens and cellular debris. As oxidative stress increases, the function of alveolar macrophages may become compromised, leading to decreased ability to clear pathogens and cellular damage [6,19]. The pathogenesis of BPD has been associated with the release of various inflammatory factors by alveolar macrophages. Pro-inflammatory cytokines, such as IL-1β, contribute to impaired alveolarization in affected individuals [20]. Therefore, to investigate the protective mechanism of exosomes against BPD, hyperoxia-induced mouse monocyte macrophages were used to simulate the BPD microenvironment.

In this study, exosomes were isolated from the supernatant of UCMSC through ultracentrifugation. The surface markers of hUCMSC-Exos were identified through Western blotting analysis, and the exosomes were labeled with PKH26 red fluorescent dye for co-culture with RAW264.7 macrophages. The results confirmed that exosomes were internalized by macrophages across various experimental conditions.

Nigericin, known to induce apoptosis and pyroptosis in mammals, was employed in this study to induce cellular injury. CCK-8-assays demonstrated that hUCMSC-Exos were effective in inhibiting the reduction in cell viability induced by Nigericin and hyperoxia. The NLRP3 inflammasome plays a crucial role in the regulation of macrophage pyroptosis, which has been implicated in the pathogenesis of lung injury [21]. Our study found the expression levels of NLRP3 ASC and caspase-1 levels were elevated in the Hyperoxia group compared to the Normoxia group, indicating a heightened activation of the NLRP3 inflammasome under hyperoxic conditions. In contrast, the expression of NLRP3, ASC and caspase-1 was decreased in the Hyperoxia + hUCMSC-Exos group, confirming that hUCMSC-Exos could reverse hyperoxia-induced NLRP3 inflammasome expression in lung macrophages and protect against associated damage.

Hou *et al*. found that hUCMSC-Exos could improve abnormal elastin expression in hyperoxia-induced BPD models, thereby protecting against lung injury [22]. Similarly, Xiong *et al*. discovered that intraperitoneal injection of large amounts of hUCMSC-Exos could effectively treat hyperoxia-induced BPD in newborn rats [23]. Our findings are consistent with these previous reports, further corroborating the potential protective effects of hUCMSC-Exos in BPD.

Moreover, in the Hyperoxia + hUCMSC-Exos group, inflammasome-related proteins NLRP3, ASC and caspase-1 were upregulated compared to the Hyperoxia + hUCMSC-Exos + Nigericin group. Similar observations have been made in other studies, such as those by Liu *et al*., who found that emodin alleviated LPS-induced acute lung injury by inhibiting the NLRP3 inflammasome-dependent pyroptosis signaling pathway [24]. Yang *et al*. also demonstrated that the COX-2/s EH dual inhibitor PTUPB reduced LPS-induced acute lung injury in mice by inhibiting NLRP3 inflammasome activation [25]. Xu *et al*. confirmed that knocking out the NLRP3 gene had a protective effect against lung injury caused by cerebral ischemia-reperfusion [26].

In recent years, scholars have found that endoplasmic reticulum stress is a cellular response that ensures the correct folding of proteins [12]. It is initiated when sensing inflammatory infections and participates in the occurrence and development of lung injury by regulating immune recognition, macrophage activation, and alveolar endothelial function [27,28]. Overactivation of endoplasmic reticulum stress leads to cytoplasmic release of calcium ions from the endoplasmic reticulum, resulting in NLRP3 inflammasome activation and mitochondrial dependent death [29]. This study found that compared with the Normoxia group, ATF4 was highly expressed in the Hyperoxia group; Compared with the Hyperoxia group, the expression of ATF4 was low in the Hyperoxia + hUCMSC-Exos group; Compared with the Hyperoxia + hUCMSC-Exos group, the expression of ATF4 was increased in the Hyperoxia + hUCMSC-Exos + Nigericin group, indicating that in the model of lung and bronchial injury induced by Hyperoxia and Nigericin, the expression of ATF4 was increased due to endoplasmic reticulum stress. After adding HucMSC-Exos, the expression of ATF4 decreased and endoplasmic reticulum stress was improved, suggesting that ATF4 is involved in the occurrence and development of BPD, while HucMSC-Exos can inhibit endoplasmic reticulum stress.

Previous studies have shown that hUCMSC-Exos have anti-inflammatory effects [30]. IL-33, IL-6, IL-1β, TNF-α, and IL-18 are important pro-inflammatory factors in the body that can stimulate surrounding cells and further enhance the inflammatory response [8]. Further research in this study found that hUCMSC-Exos can regulate the release of inflammatory factors. Compared with the Normoxia group, the concentrations of cytokines IL-33, IL-6, IL-1 β, TNF-α, and IL-18 increased in the Hyperoxia group. Compared with the Hyperoxia group, the concentrations of cytokines IL-33, IL-6, IL-1β, TNF-α, and IL-18 decreased in the Hyperoxia + hUCMSC-Exos group, indicating that hUCMSC-Exos reduced the activity of inflammatory factors in lung injury.

## Conclusions

In summary, hUCMSC-Exos reduces the expression of endoplasmic reticulum stress-related protein ATF4, alleviates high oxygen induced endoplasmic reticulum stress, and inhibits macrophage pyroptosis by regulating the expression of NLRP3 inflammasome related proteins (NLRP3, ASC, caspase-1). In addition, hUCMSC-Exos significantly reduces the secretion levels of inflammatory factors (IL-33, IL-6, IL-1β, TNF-α, IL-18), indicating its significant anti-inflammatory effect. These findings suggest that hUCMSC-Exos may exert their protective effects through multi-target synergistic effects, which may have potential benefits for BPD. This provides a new perspective on the potential protective mechanisms of HucMSC-Exos in bronchopulmonary dysplasia (BPD). However, this study is limited to *in vitro* experiments, and its results need to be further validated in *in vivo* animal models.

## Figures and Tables

**Table 1:** Cell seeding details.

*Vessel Type*	Culture Medium	Cell Count
*96-well plate*	100 μl	1×10^5^
*25 T culture flask*	5 ml	2.5×10^5^
*Confocal dish*	2 ml	9×10^5^

**Table 2:** Primer record table.

*Gene name*		Sequence (5′-3′)
*NlRP3*	F	TCTGCACCCGGACTGTAAAC
	R	CATTGTTGCCCAGGTTCAGC
*ASC*	F	TGAGCAGCTGCAAACGACTA
	R	TGTGCTGGTCCACAAAGTGT
*Caspase-1*	F	ACTGACTGGGACCCTCAAGT
	R	GCAAGACGTGTACGAGTGGT
*ATF4*	F	GCAAAGCCCCACAACATGAC
	R	GCTTGGCCACCTCCAGATAG
*Actin*	F	TTACAGGAAGTCCCTCACCC
	R	ACACAGGAAGCAATGCTGTCAC

**Table 3:** Antibody dilution information.

*Antibody name*	Dilution ratio
*NlRP3*	1:1000
*ASC*	1:1000
*Caspase-1*	1:1000
*Il-1β*	1:5000
*Il-18*	1:1000
*ATF4*	1:1000
*IgG*	1:5500
*β-actin*	1:6500

## Abbreviations

4-PBA: 4-Phenylbutyric acid
ATF4: Activating Transcription Factor 4
ASC: apoptosis-associated speck-like protein containing a CARD
BCA: Bicinchoninic Acid Assay
CCK-8: Cell Counting Kit-8
CHOP: C/EBP Homologous Protein
DMEM: Dulbecco’s Modified Eagle Medium
ERO-1α: Endoplasmic Reticulum Oxidoreductase 1 Alpha
FBS: Fetal Bovine Serum
GADD34: Growth Arrest and DNA Damage-Inducible 34
hUCMSC-Exos: Human umbilical cord mesenchymal stem cell-derived exosomes
LPS: Lipopolysaccharide
NLRP3: NOD-like receptor thermal protein domain associated protein 3
NTA: Nanoparticle Tracking Analysis
PBS: Phosphate Buffered Saline
PBST: Phosphate-Buffered Saline with Tween-20
qRT-PCR: Quantitative Real-Time Polymerase Chain Reaction
SDS-PAGE: Sodium Dodecyl Sulfate-Polyacrylamide Gel Electrophoresis
